# Cornelia de Lange syndrome mutations in NIPBL can impair cohesin-mediated DNA loop extrusion

**DOI:** 10.1073/pnas.2201029119

**Published:** 2022-04-27

**Authors:** Melanie Panarotto, Iain F. Davidson, Gabriele Litos, Alexander Schleiffer, Jan-Michael Peters

**Affiliations:** ^a^Research Institute of Molecular Pathology, Vienna BioCenter, 1030 Vienna, Austria;; ^b^Vienna BioCenter PhD Program, Doctoral School of the University of Vienna and Medical University of Vienna, A-1030 Vienna, Austria

**Keywords:** cohesin, Cornelia de Lange syndrome, developmental disorder, DNA loop extrusion, NIPBL

## Abstract

Cornelia de Lange syndrome (CdLS) is a developmental multisystem disorder frequently associated with mutations in NIPBL. CdLS is thought to arise from developmental gene regulation defects, but how NIPBL mutations cause these is unknown. Here we show that several NIPBL mutations impair the DNA loop extrusion activity of cohesin. Because this activity is required for the formation of chromatin loops and topologically associating domains, which have important roles in gene regulation, our results suggest that defects in cohesin-mediated loop extrusion contribute to the etiology of CdLS by altering interactions between developmental genes and their enhancers.

Cornelia de Lange syndrome (CdLS) (Online Mendelian Inheritance in Man entries 122470, 300590, 300882, 610759, and 614701) is characterized by physical, cognitive, and behavioral traits, including neurodevelopmental defects, facial dysmorphism, and upper-limb abnormalities (1). About 70% of CdLS patients carry heterozygous and sometimes mosaic mutations in NIPBL (2, 3). NIPBL is a 316-kDa protein, which associates with cohesin, a multisubunit ATPase complex of the structural maintenance of chromosomes (SMC) family (4). Cohesin is required for sister chromatid cohesion, DNA damage repair, and folding of chromatin fibers into loops and topologically associating domains (TADs) (4). NIPBL has been proposed to load cohesin onto DNA (reviewed in ref. 4), stimulates cohesin’s ATPase activity (5, 6), is, like cohesin, required for chromatin looping and TAD formation (7), and is essential for cohesin’s ability to extrude DNA into loops (5, 6). This loop extrusion depends on cohesin’s ATPase activity and is thought to be the process through which chromatin loops and TADs are formed (4).

Mutations in other cohesin regulators and subunits have also been identified in CdLS patients (∼10% of cases), suggesting that NIPBL mutations contribute to CdLS by affecting cohesin functions (1, 8). Most NIPBL mutations and a subset of the cohesin mutations are associated with a classical spectrum of CdLS features. In atypical patients with CdLS-like symptoms, mutations in chromatin and gene regulation proteins have been identified (8). These findings and the observation that patient-derived cells and CdLS animal models show alterations in their transcriptomes suggest that CdLS is caused by developmental gene regulation defects (4, 9, 10). However, how NIPBL and cohesin mutations lead to such defects is unknown.

## Results and Discussion

**Fig. 1.**
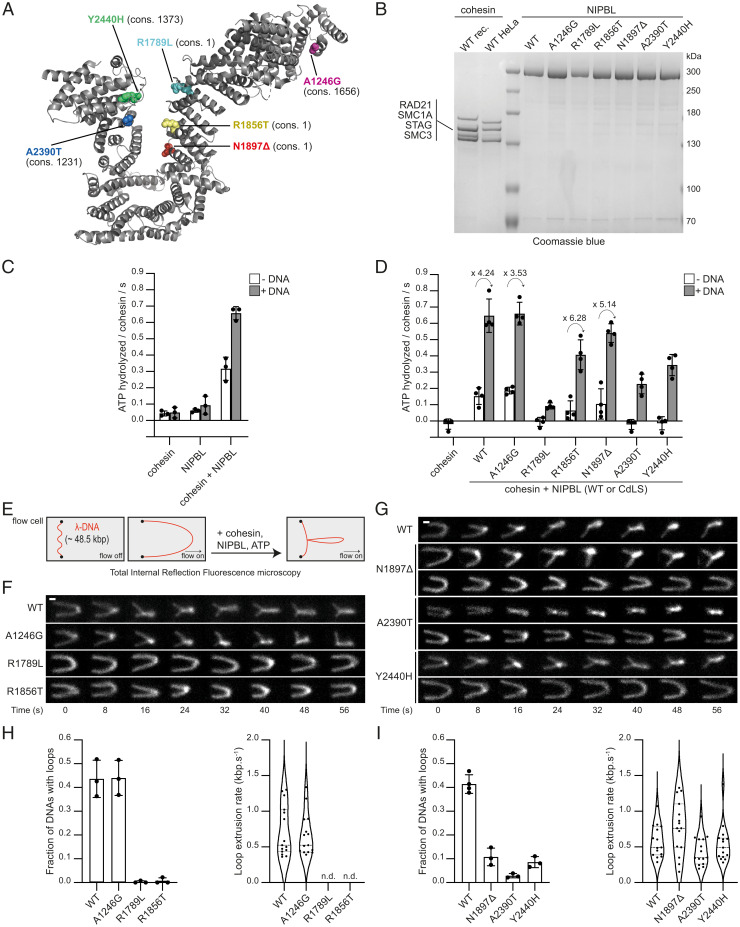
CdLS mutations in NIPBL can impair cohesin’s ATPase and DNA loop extrusion activities. (*A*) Structure of NIPBL (Protein Data Bank ID: 6wg3.E). Residues 1193 to 2628 are visible. Mutated residues are indicated as spheres. The evolutionary conservation ranks (cons.) among vertebrate orthologs are indicated in brackets: range 1 to 2804; 1, most conserved; 2804, least conserved. (*B*) Coomassie staining of cohesin and NIPBL after sodium dodecyl sulfate polyacrylamide gel electrophoresis. Subunits of recombinant (rec.) and HeLa cohesin are indicated. (*C* and *D*) Cohesin ATPase rates (mean ± SD of three and four independent experiments, for *C* and *D*, respectively) in the presence of the indicated components. The fold stimulation of ATPase activities by DNA is indicated above the arrows. (*E*) Cartoon illustration of the loop extrusion assay. (*F* and *G*) Stills from time-lapse recordings of representative DNA molecules in the presence of cohesin, ATP, and wild-type (WT) or mutant NIPBL. DNA was stained by Sytox Orange. (Scale bar: 1 μm.) (*H* and *I*) Frequencies of DNAs with extruded loops (*Left*; mean ± SD of three independent experiments) and rates of loop extrusion (*Right*; n.d.: not determined; median and quartiles are shown).

To test whether defects in loop extrusion could contribute to CdLS, as has been discussed (4, 11), we selected six CdLS NIPBL mutations (12, 13), which represent a spectrum from high to low evolutionary conservation (Fig. 1*A*). Recombinant versions of full-length NIPBL carrying these mutations could be isolated in amounts and concentrations comparable to those of wild-type NIPBL (Fig. 1*B*), suggesting that these mutants are properly folded.

We first analyzed the ability of these mutants to stimulate cohesin’s ATPase activity. We measured this activity in the absence and presence of λ-phage DNA, which (like other DNA molecules) stimulates cohesin’s ATPase activity in the presence of NIPBL, but not in its absence (Fig. 1*C*) (4–6). NIPBL-A1246G increased cohesin’s ATPase activity as much as wild-type NIPBL in both the absence and presence of DNA (Fig. 1*D*). In contrast, in the absence of DNA, the other five mutants stimulated cohesin’s ATPase activity less (R1856T and N1897Δ) or not at all (R1789L, A2390T, and Y2440H) (Fig. 1*D*, white bars). However, in the presence of DNA, these NIPBL mutants increased cohesin’s ATPase activity to some extent, although less than wild-type NIPBL. In all cases, the fold activation of the basal cohesin–NIPBL activity by DNA was comparable (Fig. 1*D*, gray bars). These results indicate that the reduced ATPase activities of these complexes are caused by impaired abilities of the NIPBL mutants to stimulate cohesin’s ATPase activity, and not by a defect of these complexes in interacting with DNA.

To analyze the effects of these NIPBL mutations on loop extrusion, we used an in vitro reconstitution assay in which the ability of cohesin and NIPBL to reel DNA into loops can be analyzed by total internal reflection fluorescence microscopy at the single-molecule level in real time (Fig. 1*E*) (5). Loop extrusion occurred with similar frequencies and rates in the presence of wild-type NIPBL and NIPBL-A1246G (Fig. 1 *F* and *H*). In contrast, the other five NIPBL mutants only supported loop extrusion to a much lesser extent (N1897Δ, A2390T, and Y2440H) or not at all (R1789L and R1856T; Fig. 1 *F*–*I*). The few loop extrusion events that occurred in the presence of the NIPBL mutants N1897Δ, A2390T, and Y2440H occurred with rates similar to those observed with wild-type NIPBL (Fig. 1 *I*, *Right*). All NIPBL mutants that are impaired in stimulating cohesin’s ATPase activity are therefore also impaired in supporting loop extrusion, as expected given that cohesin’s ATPase activity is required for this process (5, 6). However, the opposite is not true: Although NIPBL-R1856T and NIPBL-Y2440H stimulated cohesin’s ATPase activity in the presence of DNA to a similar extent, only NIPBL-Y2440H enabled some loop extrusion. The loop extrusion defect of NIPBL-R1856T can therefore not only be explained by its reduced ATPase stimulation but must be due to additional defects.

These results show that some of the NIPBL mutations that have been identified in CdLS patients cause defects in loop extrusion and therefore suggest that deficiencies in this process can contribute to the etiology of CdLS. Importantly, our observation that DNA stimulates the ATPase activities of all mutant cohesin–NIPBL complexes shows that these can still interact with DNA (Fig. 1*D*, gray bars). These results suggest that at least some NIPBL mutations interfere with loop extrusion directly, as opposed to simply affecting the loading of cohesin onto DNA. These conclusions are supported by our recent observation that two CdLS mutations in the cohesin subunit SMC1A also impair loop extrusion but not stimulation of cohesin’s ATPase activity by DNA (SMC1A-Δ58-62 and SMC1A-R711Q; ref. 14).

The observation that NIPBL-A1246G behaved like wild-type NIPBL in our assays suggests that NIPBL mutations might also contribute to CdLS by affecting functions other than loop extrusion. Alternatively, it is possible that this mutation causes cellular loop extrusion defects, which cannot be detected in our in vitro assays because these utilize “naked” DNA and not chromatin fibers and do not contain loop extrusion boundaries and cohesin regulators as they are found in cells. It is also possible that this mutant is expressed at lower levels or is less stable than wild-type NIPBL in cells.

Defects in loop extrusion could explain how NIPBL and cohesin mutations lead to developmental gene dysregulation in CdLS patients, since some of the chromatin loops formed by this process facilitate enhancer–promoter interactions (4, 15), and since boundaries between TADs can insulate genes from activation by enhancers in other TADs (16). Consistent with this prediction, a recent study reported changes in chromatin interactions at the *H19-IGF2* locus in CdLS cells (17). Since CdLS patients carry heterozygous and sometimes mosaic mutations in *NIPBL* and cohesin genes, and since mouse models have shown that *Nipbl* heterozygosity only reduces *Nipbl* transcript levels by 30% (9), genome architecture changes in CdLS patient-derived cells are expected to be subtle. However, even small alterations in enhancer–promoter interactions could lead to the severe developmental abnormalities from which CdLS patients suffer.

## Materials and Methods

Recombinant versions of Flag-Halo-NIPBL-10xHis and cohesin [SMC1A, SMC3-Flag, RAD21(TEV)-Halo and 10xHis-STAG1] were expressed in baculovirus-infected Sf9 cells and purified by tandem-affinity chromatography via their His and Flag tags. ATPase activities of recombinant cohesin were measured by [γ-^32^P] ATP (Fig. 1*C*) or luminescence (Fig. 1*D*) detection using the ADP-Glo Kinase Assay (Promega, TM313). Cohesin purified from HeLa “Kyoto” cells expressing RAD21-Halo-Flag (5) was used with recombinant NIPBL to perform the loop extrusion experiments as described (5, 14). See *SI Appendix* for detailed protocols.

## Supplementary Material

Supplementary File

## Data Availability

All study data are included in the article and/or *SI Appendix*.
