# Supplementary material for: Cornelia de Lange syndrome mutations in NIPBL can impair cohesin-mediated DNA loop extrusion
Source: Proc Natl Acad Sci U S A. 2022 Apr 27;119(18):e2201029119. doi: 10.1073/pnas.2201029119 (PMC9170158; doi:10.1073/pnas.2201029119)
Supplement: Supplementary File [file pnas.2201029119.sapp01.pdf]

PNAS
www.pnas.org

Supplementary Information for

Cornelia de Lange syndrome mutations in NIPBL can impair cohesin-mediated DNA loop extrusion

Melanie Panarotto[a,b,1], Iain F. Davidson[a,1], Gabriele Litos[a], Alexander Schleiffer[a], and Jan-Michael Peters[a,2]

[a]Research Institute of Molecular Pathology, Vienna BioCenter, 1030 Vienna, Austria

[b]Vienna BioCenter PhD Program, Doctoral School of the University of Vienna and Medical University of Vienna, A-1030 Vienna, Austria

[1]M.P. and I.F.D. contributed equally to this work.

[2]Corresponding author: Jan-Michael Peters
Email: peters@imp.ac.at

This PDF file includes:

Extended methods
SI References

Extended methods

DNA cloning, mutagenesis and generation of bacmids

A pBig2ab plasmid (1) encoding wild-type human SMC1A, SMC3-Flag, RAD21(TEV)-Halo (with three internal tabacco etch virus (TEV) sites) and 10xHis-STAG1 subunits was used for the generation of a bacmid (2). A pLib plasmid (1) encoding wild-type human Flag-Halo-NIPBL-10xHis was used as a template for site-directed mutagenesis to introduce CdLS point mutations and for the generation of bacmids (1). The mutations were generated by polymerase chain reactions using Phusion Hotstart High Fidelity DNA Polymerase (NEB) after optimization of annealing temperatures. The primers for mutagenesis were designed by NEBaseChanger (https://nebasechanger.neb.com) and by the codon optimization tool from IDT (https://eu.idtdna.com/pages/tools/codon-optimization-tool). The selected amplicon was digested by DpnI (Thermo Fisher) at 37 °C for at least 1 h. The digested and purified amplicon was phosphorylated by T4 polynucleotide kinase (NEB, 10,000 U/ml) at ~ 0.17 U/μl and ligated by T4 ligase (NEB, 2,000,000 U/ml) at ~ 33 U/μl, in T4 ligase buffer (NEB), with 0.5 – 1 h incubation at 37 °C. This resulting amplicon was then transformed in DH5α bacteria for subsequent clonal amplification. The results of the mutagenesis experiments were analyzed by Sanger DNA sequencing of the entire NIPBL sequence to confirm the presence of the desired mutation and to rule out off-target effects. pBig2ab and pLib (wild-type or CdLS) plasmids were transformed into DH10 Multibac bacteria to generate bacmid DNA as previously described (3).

Expression of recombinant proteins

Sf9 insect cells were transfected with bacmids by lipofection (FuGENE 6 Transfection Reagent, Promega) and cultured for 96 h at 27 °C to generate a V0 Baculovirus stock. A culture of Sf9 insect cells (50 ml of 1 million cells per milliliter) was infected with ~ 3 ml of V0 Baculovirus stock and cultured for ~ 3 days, at 100 rpm and 27 °C in Grace medium to generate a V1 Baculovirus stock, which was used for protein expression. All protein expression cultures were infected with Baculovirus at a density of 1 million cells per milliliter, and cultured at 100 rpm and 27 °C in Grace medium in baffled 2 L flasks filled to 750 ml. Cultures were harvested after 48 – 60 h expression; protein expression (V0, V1, and final expression) was monitored by the expression of a fluorescent marker encoded by the bacmid. Harvested cultures were washed in cold PBS, frozen in liquid nitrogen, and stored at -80 °C.

Purification of recombinant proteins

Proteins were purified as previously described (2). All steps were performed at 4 °C unless indicated otherwise. Sf9 insect cell pellets (~ 30 ml) were thawed and lysed by Dounce homogenization (30 strokes) in ~ 70 ml buffer A (25 mM NaH_2PO_4/Na_2HPO_4 pH 7.5, 500 mM NaCl, 5 % glycerol) supplemented with 10 mM imidazole pH 7.5, 0.05 % Tween 20, 1 mM PMSF, 3 mM beta-mercaptoethanol, 10 μg/ml aprotinin, 2 mM benzamidine (Sigma) and cOmplete EDTA-free protease inhibitor cocktail (Merck, 11873580001). After centrifugation (19,500 rpm, 45 min), the soluble fraction was combined with Toyopearl AF-chelate-650M resin (Tosoh Bioscience) pre-charged with Ni^{2+} ions and stirred for 3 h. Beads were washed with 3 x 10 volumes of buffer A supplemented with 15 mM imidazole, pH 7.5, 0.01 % Tween 20, 1 mM PMSF, 2 mM benzamidine. Bound proteins were eluted with 25 ml buffer B (25 mM NaH_2PO_4/Na_2HPO_4 pH 7.5, 150 mM NaCl, 5 % glycerol) supplemented with 300 mM imidazole pH 7.5, 0.05 % Tween 20, 1 mM PMSF, and 2 mM benzamidine. The eluate was combined with 4 ml of FLAG-M2 agarose resin (Sigma, A2220) and stirred for 3 h. Beads were washed with 3 volumes of buffer C (25 mM NaH_2PO_4/Na_2HPO_4 pH 7.5, 150 mM NaCl, 5 % glycerol, 50 mM imidazole pH 7.5). Bound proteins were eluted with 15 ml buffer C supplemented with 0.5 mg/ml 3xFlag peptide. Eluates were concentrated to ~ 0.3 ml using Vivaspin 20-50 kDa MWCO ultrafiltration units (Sartorius, VS2042), frozen in liquid nitrogen and stored at -80 °C. Purity, stoichiometry and concentration of the protein solution were determined by SDS-PAGE.

Purification of cohesin from HeLa cells

Cohesin complexes containing SMC1A, SMC3, RAD21 and either STAG1 or STAG2 (4) were purified from a HeLa "Kyoto" cell line expressing RAD21-Halo-Flag (2) as previously described (2). All steps were performed at 4 °C unless indicated otherwise. Cell pellets (~ 8 ml) were thawed and resuspended in 80 ml buffer D (20 mM Tris pH 7.5, 1.5 mM $MgCl_2$,10 mM KCl) supplemented with 1 mM PMSF and cOmplete EDTA-free protease inhibitor cocktail (Merck, 11873580001). Cells were lysed by Dounce homogenization

(10 strokes), incubated on ice for 10 min and then Dounce homogenized another 10 times. Nuclei were pelleted by centrifugation (2000 rpm, 10 min). Pelleted nuclei were resuspended in 36 ml buffer D supplemented as above, and Dounce homogenized. NaCl was added dropwise to a final concentration of 500 mM while stirring for 10 min. Subsequently, Tween 20 was added dropwise to a final concentration of 0.1 % while stirring for 10 min. The lysate was sonicated (Branson Digital Sonifier; 3 x 20 s cycles of 0.4 s pulses at 60 % amplitude, followed by 1.5 s pause). After centrifugation (18,500 rpm, 30 min), the soluble fraction was combined with FLAG-M2 agarose resin (Sigma, A2220) and stirred for 3 h. Beads were washed with 10 volumes of buffer E (25 mM NaH_2PO_4/Na_2HPO_4 pH 7.5, 500 mM NaCl, 5 % glycerol, 1 mM EDTA) and 3 x with 10 volumes of buffer F (25 mM NaH_2PO_4/Na_2HPO_4 pH 7.5, 150 mM NaCl, 5 % glycerol, 1 mM EDTA). Bound proteins were eluted with 12 ml buffer F supplemented with 0.5 mg/ml 3xFlag peptide. Eluates were concentrated to ~ 0.2 ml using Vivaspin 2, 50 kDa MWCO ultrafiltration (Sartorius, VS0201), frozen in liquid nitrogen and stored at -80 °C. Purity, stoichiometry, and concentration of the protein solution were determined by Coomassie staining after SDS-PAGE.

ATPase assays by luminescence detection
The ability of wild-type and mutant NIPBL to stimulate cohesin's ATPase was determined using the ADP-Glo Kinase Assay (Promega, TM313). Recombinant cohesin (25 nM) was incubated, where indicated, with 50 nM NIPBL, with or without 10 ng/µl λ-DNA (NEB, N3011S), in ATPase reaction buffer (final composition: 20 mM NaH_2PO_4/Na_2HPO_4 pH 7.5, 50 mM NaCl, 2.5 mM $MgCl_2$, 1 mM DTT, 0.75 % glycerol, 7.5 mM imidazole, 0.1 mg/ml BSA, 0.3 mM EDTA, 1 mM ATP). The reactions were incubated at 37 °C for 1 h and stopped by transferring the samples on ice. The remaining ATP was depleted by the addition of ADP-Glo Reagent; Kinase Detection Reagent was added to convert ADP to ATP and allow the newly synthesized ATP to be measured using a luciferase/luciferin reaction. Three independent experiments showed that at this 1 h time point ATPase activity increased linearly with time. The solutions were transferred into a 96 well white plate (Corning) for luminescence detection using a PheraStar FX plate reader (BMG Lab tech) and PheraStar Mars software. Three measurements per sample at 30 s intervals were performed. ATP hydrolysis detected in the buffer not containing protein or DNA was subtracted from the values measured in presence of proteins ± DNA. Activities were calculated using a standard curve obtained by measuring samples containing a dilution series of ATP and ADP as described in the ADP-Glo Kinase Assay manual and plotted using Prism (GraphPad).

ATPase assays by [γ-^{32}P] ATP detection
The assay was performed as previously described (2). Recombinant cohesin or NIPBL or both were incubated at 60 nM each in ATPase reaction buffer (final composition: 25 mM NaH_2PO_4/Na_2HPO_4 pH 7.5, 50 mM NaCl, 2.5 mM $MgCl_2$, 1 mM DTT, 0.2 % glycerol, 2 mM imidazole pH 7.5, 0.1 mg/ml BSA, 2 mM ATP and 10 nM [γ-^{32}P] ATP (Hartmann Analytic, SCP-501)). Where indicated, reactions were supplemented with 20 ng/µl λ-DNA (NEB, N3011S). Reactions were incubated at 37 °C for 3, 10, 20, 30, and 40 minutes and stopped by adding 1 % SDS and 10 mM EDTA. Reaction products were separated on polyethyleneimide plates (Sigma, 1055790001) by thin-layer-chromatography using 0.75 M KH_2PO_4 (pH 3.4) and analyzed by phosphor imaging with a Typhoon Scanner (GE Healthcare) and quantified with ImageJ. The percentage of ATP hydrolysed at each timepoint was determined and subtracted by the buffer only control value. Data was plotted in Prism (GraphPad) and linear regression was used to determine ATPase rates.

DNA loop extrusion TIRF microscopy assay and image analysis
The assay was performed as previously described (2, 5). In brief, microfluidic flow cells were prepared with two inlets and two outlets to allow perpendicular changes in the direction of flow. Flow cells were incubated with 1 mg/ml Avidin DN (Vector Laboratories, A-3100-1) for 15 min, then washed with DNA buffer (20 mM Tris pH 7.5, 150 mM NaCl, 0.25 mg/ml BSA (Thermo Fisher, AM2616). 40 µl of 22.5 pM doubly biotinylated λ-DNA (NEB, N3011S) was introduced into flow cells at 5 µl/min in DNA buffer, supplemented with 20 nM Sytox Orange (Thermo Fisher, S11368). Flow cells were washed with 20 µl of DNA wash buffer (50 mM Tris pH 7.5, 200 mM NaCl, 1 mM $MgCl_2$, 5 % glycerol, 0.25 mg/ml BSA, 1 mM DTT, 20 nM Sytox Orange) at 5 µl/min. Flow cells were then switched to perpendicular flow and washed with 0.5 ml DNA wash buffer at 100 µl/min. Subsequently, 0.4 ml of imaging buffer (50 mM Tris pH 7.5, 50 mM NaCl, 2.5 mM $MgCl_2$, 0.25 mg/ml BSA, 0.05 % Tween, 20 nM Sytox Orange) was flowed-in twice at 200 µl/min. 0.1 ml of imaging

buffer supplemented with an oxygen scavenging system (0.2 mg/ml glucose oxidase (Sigma, G2133), 35 µg/ml catalase (Sigma, C-40), 9 mg/ml β-D-glucose, 2 mM Trolox (Sigma, 238813), 1 mM DTT, 250 nM Sytox Orange, and 5 mM ATP (Jena Biosciences, NU-1010) was flowed-in at 100 µl/min. Proteins were then introduced into flow cells at 30 µl/min in 0.25 ml imaging buffer supplemented as above. HeLa cohesin and recombinant NIPBL were introduced into flow cells at a final concentration of 5 nM and 15 nM, respectively. Experiments were performed at 37 °C. Time-lapse microscopy images were acquired at 4 s intervals using a Zeiss TIRF 3 Axio Observer setup. 561 nm laser was used to excite Sytox Orange. Image analysis was performed in ImageJ. To determine the fraction of DNA molecules that were extruded into loops, the number of doubly tethered DNAs that formed loops during the 8 min 20 s protein flow-in time period was divided by the total number of doubly tethered DNAs that were stretched by perpendicular flow. DNAs that were singly-tethered or oriented in such a way that loops were obscured were excluded from analysis.

To determine the rate of LE, a custom ImageJ script was used to measure the length of DNA molecules before LE and the length of DNA not contained within the loop during LE. These measurements were converted into kbp, plotted as a function of time, and used to calculate the LE rate. Fifteen representative loops were analyzed per condition. Prism (GraphPad) was used to show the TIRF assay results.

Sequence alignments, conservation scores and ranks

NIPBL orthologs were collected in the UniProt reference proteome database in NCBI-blast searches with full-length human NIPBL (sp|Q6KC79|NIPBL_HUMAN) or budding yeast Scc2 (sp|Q04002|SCC2_YEAST) as query, applying highly significant e-values (< 1e-20) (6, 7). Three sequence sets were compiled with a specificity for vertebrates, animals and eukaryotes (including fungi, animal, plant, red algae, alveolates and kinetoplastids). Sequences were aligned with mafft (-linsi option, v7.427; (8)), the conservation scores determined with AACon (v.1.1, KARLIN method; https://www.compbio.dundee.ac.uk/aacon/) and referenced to human NIPBL (length 2,804 amino acids). The position-specific conservation scores were ranked in descending order using the RANK.EQ function in Microsoft-Excel. If there are positions with equal conservation score, the top rank of that set of values is returned. The structure of NIPBL was visualized in PyMol (Schrödinger, L. The PyMOL Molecular Graphics System Schrödinger, LLC., Version 2.4.).

SI References

1. F. Weissmann *et al.*, biGBac enables rapid gene assembly for the expression of large multisubunit protein complexes. *Proc Natl Acad Sci U S A* **113**, E2564-2569 (2016).
2. I. F. Davidson *et al.*, DNA loop extrusion by human cohesin. *Science* **366**, 1338-1345 (2019).
3. F. Weissmann, J. M. Peters, Expressing Multi-subunit Complexes Using biGBac. *Methods Mol Biol* **1764**, 329-343 (2018).
4. I. Sumara, E. Vorlaufer, C. Gieffers, B. H. Peters, J. M. Peters, Characterization of vertebrate cohesin complexes and their regulation in prophase. *J Cell Biol* **151**, 749-762 (2000).
5. B. W. Bauer et al., Cohesin mediates DNA loop extrusion by a "swing and clamp" mechanism. Cell 184, 5448-5464.e5422 (2021).
6. UniProt Consortium, UniProt: the universal protein knowledgebase in 2021. *Nucleic Acids Res* **49**, D480-d489 (2021).
7. S. F. Altschul *et al.*, Gapped BLAST and PSI-BLAST: a new generation of protein database search programs. *Nucleic Acids Res* **25**, 3389-3402 (1997).
8. K. Katoh, H. Toh, Recent developments in the MAFFT multiple sequence alignment program. *Brief Bioinform* **9**, 286-298 (2008).
